# Supplementary material for: Adaptive Phage Therapy for the Prevention of Recurrent Nosocomial Pneumonia: Novel Protocol Description and Case Series
Source: Antibiotics (Basel). 2023 Dec 14;12(12):1734. doi: 10.3390/antibiotics12121734 (PMC10741035; doi:10.3390/antibiotics12121734)
Supplement: Supplementary file 1 [file antibiotics-12-01734-s001.zip › antibiotics-2684714-supplementary.pdf]

Table S1. Antibiotic sensitivity and resistance markers of K. pneumoniae and A. baumannii isolated from patient 92/23's bronchoalveolar lavage sample. Determination of antibiotic sensitivity was performed on a BD Phoenix-100 automated bacteriological analyzer (BDBiosciences, San Jose, CA, USA) and data interpretation was conducted according to the installed EUCAST protocols.

**Antibiotic**	***K. pneumoniae***		***A. baumannii***	
	**Susceptibility**	**MIC**	**Susceptibility**	**MIC**
Amikacin	R	16 mg/L	R	>32 mg/L
Amoxicillin/Clavulanate	R	>32/2 mg/L	-	-
Ampicillin	R	>16 mg/L	-	-
Gentamicin	S	≤2 mg/L	R	>8 mg/L
Imipenem	R	>8 mg/L	R	>8 mg/L
Levofloxacin	R	>4 mg/L	R	>2 mg/L
Piperacillin/Tazobactam	R	>32/4 mg/L	-	-
Tobramycin	R	>8 mg/L	-	-
Trimethoprim/ Sulfameth-oxazole	S	≤2/38 mg/L	R	>8/152 mg/L
Phosphomycin	S	≤16 mg/L	-	-
Cefazolin	R	>32 mg/L	-	-
Ceftazidime	R	>16 mg/L	-	-
Ceftriaxone	R	>4 mg/L	-	-
Ciprofloxacin	R	>1 mg/L	R	>1 mg/L
Ertapenem	R	>2 mg/L	-	-
Meropenem	R	>8 mg/L	R	>8 mg/L
Resistance marker	Extended-spectrum beta-lactamase (ESBL)		Carbapenemase producer (CARB)	
	Potential carbapenemase producer (ALERT1)		-	

MIC—minimum inhibitory concentration; R—resistant; S—sensitive.

Table S2. Antibiotic sensitivity and resistance markers of K. pneumoniae, A. baumannii and P. aeruginosa isolated from patient 62/22's bronchoalveolar lavage sample. Determination of antibiotic sensitivity was performed on a BD Phoenix-100 automated bacteriological analyzer (BDBiosciences, USA) and data interpretation was conducted according to the installed EUCAST protocols.

**Antibiotic**	***A. baumannii***		***K. pneumoniae***		***P. aeruginosa***	
	**Susceptibility**	**MIC**	**Susceptibility**	**MIC**	**Susceptibility**	**MIC**
Amikacin	R	>32 mg/L	S	≤8 mg/L	S	16 mg/L
Gentamicin	R	>8 mg/L	R	>8 mg/L	-	-
Imipenem	R	>8 mg/L	R	>8 mg/L	R	>8 mg/L
Levofloxacin	R	>2 mg/L	R	>2 mg/L	R	>2 mg/L
Meropenem	R	>8 mg/L	R	>8 mg/L	R	>8 mg/L
Trimethoprim/ Sulfamethoxazole	S	<2/38 mg/L	S	<2/38 mg/L	-	-
Ciprofloxacin	R	>1 mg/L	R	>1 mg/L	R	>1 mg/L
Piperacillin/ Tazobactam	-	-	R	>16/4 mg/L	R	>16/4 mg/L
Cefepime	-	-	R	>8 mg/L	R	>8 mg/L
Ceftazidime	-	-	R	>8 mg/L	R	>8 mg/L
Cefuroxime	-	-	R	>16 mg/L	-	-
Amoxicillin/ Clavulanate	-	-	R	>16/2 mg/L	-	-
Ampicillin	-	-	R	>16 mg/L	-	-
Tigecycline	-	-	S	2 mg/L	-	-
Cefazolin	-	-	R	>32 mg/L	-	-
Ceftriaxone	-	-	R	>4 mg/L	-	-
Ertapenem	-	-	R	>1 mg/L	-	-
Resistance marker	-		Extended-spectrum beta-lactamase (ESBL)		-	
	-		Potential carbapenemase producer (ALERT1)		-	

MIC—minimum inhibitory concentration; R—resistant; S—sensitive.

Table S3. Antibiotic sensitivity and resistance markers of S. aureus isolated from patient 62/22's bronchoalveolar lavage sample. Determination of antibiotic sensitivity was performed on a BD Phoenix-100 automated bacteriological analyzer (BDBiosciences, USA) and data interpretation was conducted according to the installed EUCAST protocols.

**Antibiotic**	***S. aureus***		**Antibiotic**	***S. aureus***	
	**Susceptibility**	**MIC**		**Susceptibility**	**MIC**
Levofloxacin	R	>4 mg/L	Rifampin	R	>2 mg/L
Ciprofloxacin	R	>4 mg/L	Teicoplanin	S	≤0.5 mg/L
Mupirocin	I	≤256 mg/L	Tetracycline	R	>2 mg/L
Vancomycin	S	1 mg/L	Tigecycline	S	0.5 mg/L
Gentamicin	R	>4 mg/L	Tobramycin	R	>4 mg/L
Daptomycin	S	1 mg/L	Trimethoprim/ Sulfa-methoxazole	S	<0.5/9.5 mg/L
Clindamycin	R	>1 mg/L	Phosphomycin	S	≤16 mg/L
Linezolid	S	2 mg/L	Fucidin	S	≤0.5 mg/L
Moxifloxacin	R	>2 mg/L	Quinupristin/ Dal-fopristin	S	≤0.5 mg/L
Oxacillin	R	>2 mg/L	Chloramphenicol	R	>16 mg/L
Penicillin	R	>0.25 mg/L	Erythromycin	R	>4 mg/L
Resistance marker	Methicillin-resistant Staphylococcus aureus (MRSA)				
	MecA-mediated resistant Staphylococcus				
	Staphylococcus MLSb phenotype				

MIC—minimum inhibitory concentration; R—resistant; S—sensitive; I—intermediate.

Table S4. Antibiotic sensitivity and resistance markers of K. pneumoniae and A. baumannii isolated from patient 847/21's bronchoalveolar lavage sample. Determination of antibiotic sensitivity was performed on a BD Phoenix-100 automated bacteriological analyzer (BDBiosciences, USA) and data interpretation was conducted according to the installed EUCAST protocols.

**Antibiotic**	***K. pneumoniae***		***A. baumannii***	
	**Susceptibility**	**MIC**	**Susceptibility**	**MIC**
Amikacin	R	>32 mg/L	R	>32 mg/L
Amoxicillin/Clavulanate	R	>32/2 mg/L	-	-
Ampicillin	R	>16 mg/L	-	-
Gentamicin	R	>8 mg/L	R	>8 mg/L
Imipenem	R	>8 mg/L	S	0.5 mg/L
Levofloxacin	R	>4 mg/L	R	>2 mg/L
Piperacillin/ Tazobactam	R	>32/4 mg/L	-	-
Tigecycline	I	4 mg/L		
Tobramycin	R	>8 mg/L		
Trimethoprim/ Sulfameth-oxazole	R	>8/152 mg/L	S	≤2/38 mg/L
Phosphomycin	S	32 mg/L		
Cefazolin	R	>32 mg/L	-	-
Ceftazidime	R	>16 mg/L	-	-
Ceftriaxone	R	>4 mg/L	-	-
Ciprofloxacin	R	>1 mg/L	R	>1 mg/L
Ertapenem	R	>2 mg/L	-	-
Meropenem	R	>8 mg/L	S	1 mg/L
Resistance marker	Extended-spectrum beta-lactamase (ESBL)		-	
	Potential carbapenemase producer (ALERT1)		-	

MIC—minimum inhibitory concentration; R—resistant; S—sensitive; I—intermediate.
